# An Optimized GATK4 Pipeline for Plasmodium falciparum Whole Genome Sequencing Variant Calling and Analysis

**DOI:** 10.21203/rs.3.rs-2561857/v1

**Published:** 2023-02-14

**Authors:** Karamoko Niaré, Bryan Greenhouse, Jeffrey A Bailey

**Affiliations:** Brown University; University of California San Francisco; Brown University

## Abstract

**Background:**

Accurate variant calls from whole genome sequencing (WGS) of *Plasmodium falciparum* infections are crucial in malaria population genomics. Here we optimized a falciparum variant calling pipeline based on GATK version 4 (GATK4) and applied it to 6,626 public Illumina WGS samples.

**Methods:**

We optimized parameters that control the heterozygosity, local assembly region size, ploidy, mapping and base quality in both GATK HaplotypeCaller and GenotypeGVCFs leveraging control WGS and accurate PacBio assemblies of 10 laboratory strains. From these controls we generated a high-quality training dataset to recalibrate the raw variant data.

**Results:**

On current high-quality samples (read length = 250bp, insert size = 405 – 524 bp), we show improved sensitivity (86.6 ± 1.7% for SNPs and 82.2 ± 5.9% for indels) compared to the default GATK4 pipeline (77.7 ± 1.3% for SNPs; and 73.1 ± 5.1% for indels, adjusted P < 0.001) and previous variant calling with GATK version 3 (GATK3, 70.3 ± 3.0% for SNPs and 59.7 ± 5.8% for indels, adjusted P < 0.001). The sensitivity of our pipeline on simulated mixed infection samples (80.8 ± 6.1% for SNPs and 78.3 ± 5.1% for indels) was again improved relative to default GATK4 (68.8 ± 6.0% for SNPs and 38.9 ± 0.7% for indels, adjusted P < 0.001). Precision was high and comparable across all pipelines on each type of data tested. We further show that using the combination of high-quality SNPs and indels increases the resolution of local population population structure detection in sub-Saharan Africa. We finally demonstrate that increasing ploidy improves the detection of drug resistance mutations and estimation of complexity of infection.

**Conclusions:**

Overall, we provide an optimized GATK4 pipeline and resource for *falciparum* variant calling which should help improve genomic studies of malaria.

## Background

Malaria’s toll on human health remains unacceptably high with 627,000 deaths reported worldwide in 2020 (World Health Organization, 2021). Despite substantial progress in control made during the past two decades, decreases in mortality have slowed or reversed (World Health Organization, 2021). The vast majority of estimated deaths continue to occur in sub-Saharan Africa in children aged below 5 years due to the most virulent species, *Plasmodium falciparum*. Control efforts are challenged by the lack of highly effective vaccines, drug and insecticide resistance, failure of rapid diagnostic tests due to *hrp2* and *hrp3* deletions, and the impact of the COVID-19 pandemic (Dondorp et al., 2009; Gamboa et al., 2010; Koita et al., 2012; World Health Organization, 2021). Further progress necessitates improved tools for furthering control and for the monitoring of progress towards eventual elimination.

Whole genome sequencing (WGS) represents a key tool for genomic epidemiology and surveillance of parasite populations that circulate in malaria endemic regions. Significant advances have been made in malaria research since the first WGS data of *P. falciparum* was released in 2002 to provide a reference genome (Gardner et al., 2002). Today, laboratory and large clinical WGS datasets of parasites representing malaria infections are generated predominantly by Illumina next-generation sequencing (MalariaGEN et al., 2021). Studies based on WGS have been instrumental in defining and understanding drug resistance, including the discovery of *ketch 13 (k13)* mutations as the molecular markers of artemisinin resistance as well as providing a detailed description of the parasite population structure within and across continents (Amambua-Ngwa et al., 2019; Ariey et al., 2014; MalariaGEN et al., 2021).

A crucial step in WGS data analysis is calling variants by mapping reads onto a reference genome and looking for consistent differences representing single nucleotide polymorphisms (SNPs), insertions-deletions (indels) and structural variations. First, the genome of *P. falciparum* is AT-rich with large regions of low complexity and duplicated sequence, such as the subtelomeric regions which contain highly-related gene families. This presents a general challenge for mapping reads accurately to the reference genome. The *P. falciparum* genome is extremely repetitive in many (coding and non-coding) regions, with high frequencies of AT-rich tandem repeats that create challenges for accurate calling of variants (Hamilton et al., 2017; Miles et al., 2016). Indels are predominantly associated with short AT-rich sequence repeats and are challenging to accurately identify with short read sequencing. Consequently, SNPs, which are relatively less difficult to identify compared to indels, have been the preferred variant type for *P. falciparum* population genetics studies. Second, sequence data from natural malaria infections present an additional challenge, often containing an unknown number of clones (complexity of infection, COI) at unknown relative abundances (DePristo et al., 2006; Felger et al., 1999; Gardner et al., 2002).

Despite the fact that *P. falciparum* is entirely haploid in the human host during its lifecycle, the performance of the variant calling is affected by the high COI of clinical samples collected from patients in areas of high malaria transmission. The number of clones can sometimes reach double digits, and given their numbers and relative frequency are variable and unobserved, standard variant calling pipelines based on ploidy do not properly apply. Moreover, given the proportion of an individual clone within an infection can be extremely low, the detection of its presence and variants it may contain can be computationally challenging.

The genomic analysis toolkit (GATK) (Poplin et al., 2018; Van der Auwera & O’Connor, 2020) is a well-validated tool that has been traditionally used to call variants (SNPs and short indels) in malaria WGS data. However, this tool was initially developed for diploid human and other mammalian genomes and has not been extensively optimized for microorganisms, particularly challenging ones such as *P. falciparum*. One of the most important features of GATK is the variant recalibration step which consists of using a training dataset to score and filter out low quality variants that are more likely false than true from the raw variant data in variant call format files (VCFs). However, developing a Mendelian error-free (or error-depleted) training dataset based on sequence reads generated in the wet laboratory is currently a challenge in malaria. Additionally, GATK was designed for diploid organisms, where there are equal proportions of each chromosome present in the sample; in natural malaria infections with COI > 1 it is more difficult to distinguish the few copies of actual variants in minor clones from sequencing errors We hypothesized that this issue could be partially overcome by partitioning the ploidy until each expected variant present in the sample gets enough probability to be called.

Here we leverage the flexibility of GATK4 to optimize SNP and indel calling for improved *P. falciparum* WGS data analysis. We generate a custom variant training dataset *in silico* based on accurate PacBio assemblies of laboratory strains as templates to filter out low quality variants. We test and adjust prior variant rates and multiple parameters that control the read and mapping qualities at various stages of the pipeline. We also make a reference true callset with select known laboratory strains to evaluate the performance of our pipeline. Finally, we apply the pipeline to available public WGS data, including Malariagen’s Pf6 dataset, demonstrating improvements in population genomic analyses. Since indels have been challenging to properly call and rarely incorporated into these analyses, our study focuses on them and demonstrates their utility in increasing the resolution of population structures when combined with SNPs.

## Results

### Optimization of the pipeline on monoclonal and simulated mixed infection samples.

Towards optimizing GATK4 for *P. falciparum*, we sought to create an improved training “truth set” for the pipeline. To filter raw VCFs with a high quality truth callset, which is difficult to obtain using wet lab methods, we decided to generate a robust *in silico* training variant dataset from the PacBio assemblies (Additional file1: Figure S1). We first ran the variant calling pipeline with default settings of GATK4’s HaplotypeCaller and GenotypeGVFs tools on high-quality public Illumina read data (Additional file1: Table S1) of 10 laboratory strains (7G8, Dd2, GA01, GB4, GN01, HB3, IT, KH01, KH01 and SN01) for which there exist accurate PacBio assemblies (Otto et al., 2018; Vembar et al., 2016). We recalibrated the raw VCFs with the published training dataset generated from 3D7 × HB3, HB3 × Dd2 and 7G8 × GB4 crosses in splenectomized chimpanzees (Walliker et al. 1987; Wellems et al. 1990; Hayton et al. 2008) and used by MalariaGEN’s Pf6 release (Miles et al. 2016; MalariaGEN et al. 2021). The results were compared to reference callsets we generated from the accurate PacBio assemblies mapped onto the 3D7 reference (Methods, Additional file1: Figure S2).

**Figure 1:**
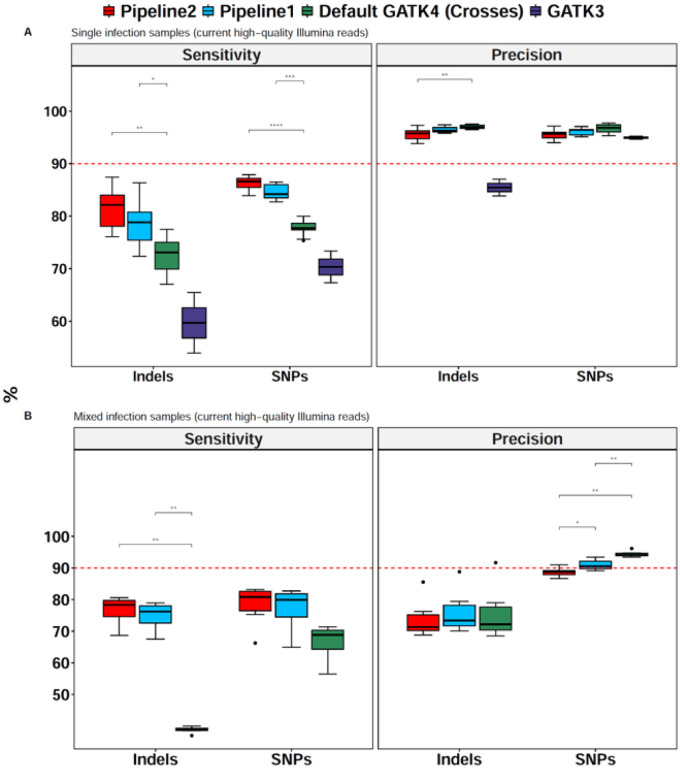
Performance of the optimized GATK4, default GATK4 and GATK3 pipelines. **A)** Pipeline performance using current high-quality Illumina read data (read length = 250 bp; insert size = 405 – 524 bp) from single infection samples.Ten laboratory strains (7G8, Dd2, GA01, GB4,GN01, HB3, IT, KH01, KH02 and SN01) were included for all the pipelines except GATK3 as only two (GN01 and KH02) of these samples were found in the GATK3 VCFs we downloaded from the MalariaGEN website. **B)** pipeline performance on simulated high-quality mixed infections samples of IT + KH01 at 95:5, 90:10, 85:15, 80:20, 75:25, and 50:50 proportions (100X read depth). Only significant statistical differences are shown (indicated by asterisks). Pipeline 1: GATK4 pipeline with default settings of HaplotypeCaller and GenotypeVCFs coupled with variant recalibration by our *in silicotraining* dataset. Pipeline2: fully optimized GATK4 pipeline with alternation of HaplotypeCaller and GenotypeGVFs parameters and variant recalibration (filtering) using our new *in silicotraining* dataset. Default GATK4 (crosses): Default GATK4 pipeline but recalibrated by the publicly available cross dataset. GATK3: same GATK3 pipeline used by MalariaGEN’s Pf6 release in which variants are recalibrated by the cross training dataset. Red dashed line represents 90% performance.

Sensitivity of this default variant calling in the core genome were 77.7 ± 1.3% (median ± interquartile range) for SNPs and 73.1 ± 5.1% for indels (Fig. 1A). When we replaced the cross dataset with our PacBio-derived *in silico* training dataset (called pipeline 1), we found greatly improved sensitivity (84.2 ± 2.5% for SNPs; and 78.8 ± 5.4% for indels). With GATK3 recalibrated with the cross training dataset, the sensitivity for both SNPs and indels was the lowest. As next step in GATK4 optimization, we altered multiple parameters of HaplotypeCaller and GenotypeGVFs of pipeline 1 to make pipeline 2, including adjusting expected SNP and indel rates (--heterozygosity and -indel-heterozygosity) and parameters that control base quality (--base-quality-score-threshold and -stand-call-conf), mapping (-mbq and -DF) and local assembly size (--min-assembly-region-size), aiming to further improve the sensitivity and reduce false calls. While default values of pipeline 1 are fairly robust, sensitivity increased significantly to 86.6 ± 1.7% for SNPs and 82.2 ± 5.9% for indels when modified parameters were used (Methods; Fig. 1A; adjusted P < 0.001, Wilcoxon test, Pipeline 2 vs. default GATK4 with cross training dataset for both SNPs and Indels). Despite the trade off between sensitivity and precision, the latter was very high (> 90%) for all GATK4 pipelines, including our pipeline 1 and 2.

We finally tested the GATK4 pipelines on simulated mixed infection samples after combining high-quality reads of IT + KH01 at different proportions (95:5, 90:10, 85:15, 80:20, 75:25 and 50:50) to make 100X coverage. Sensitivity of our optimized pipelines was higher than that of the default GATK4 pipeline trained with the cross dataset for both SNPs and indels (adjusted P = 0.001, Wilcoxon test) (Fig. 1B). Interestingly, the most significant performance gains were observed with indels for which the sensitivity of pipeline 2 was 78.3 ± 5.1% versus 38.9 ± 0.7% for default GATK4 with cross training dataset (Fig. 1B, adjusted P < 0.001, Wilcoxon test). Here the precision was similar across all GATK4 pipelines but lower compared to single infection samples.

**Figure 2:**
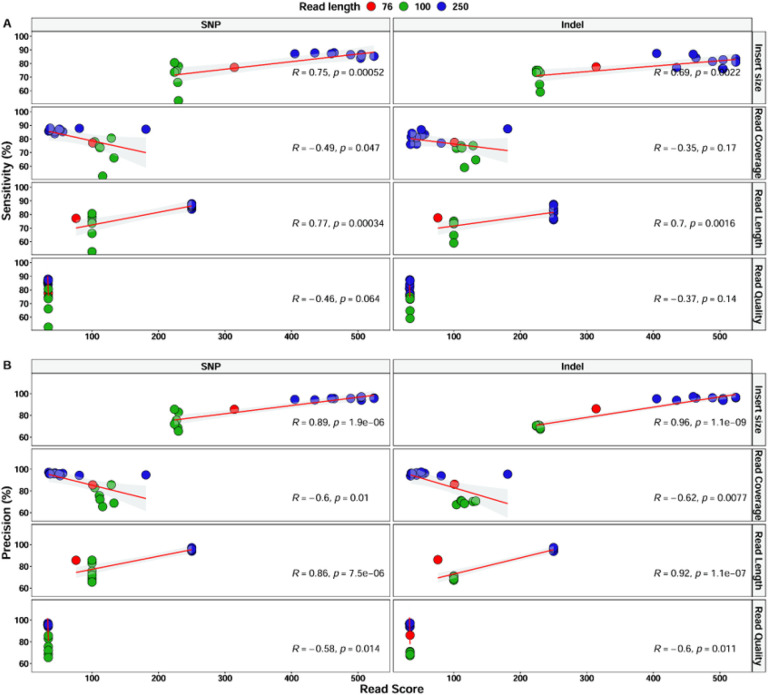
Correlation analysis between sequencing quality parameters and variant calling performance, sensitivity **(A)** and precision **(B)**. Red (75bp) and green (100bp) represent samples with old shorter Illumina reads. Samples with current longer Illumina reads are colored blue. Read score in the x-axis represents values for either insert size, read coverage, read length or read quality. Pearson correlation was used.

To understand how sequencing quality affects the results of our pipelines, we added shorter (old) Illumina read samples (n = 7) from the Pf6 dataset and analyzed any potential effect of the read coverage, insert size, read length and read quality on variant calling performance (Additional file1: Table S2). In spite of the great variation in the read coverage (median between 35 and 180X), neither the specificity nor the precision was correlated with this parameter (Fig. 2). Interestingly, we found a strong positive correlation between pipeline performance and insert size and read length (Fig. 2).

### Combination of SNPs and indels shows higher resolution of population structure

**Figure 3:**
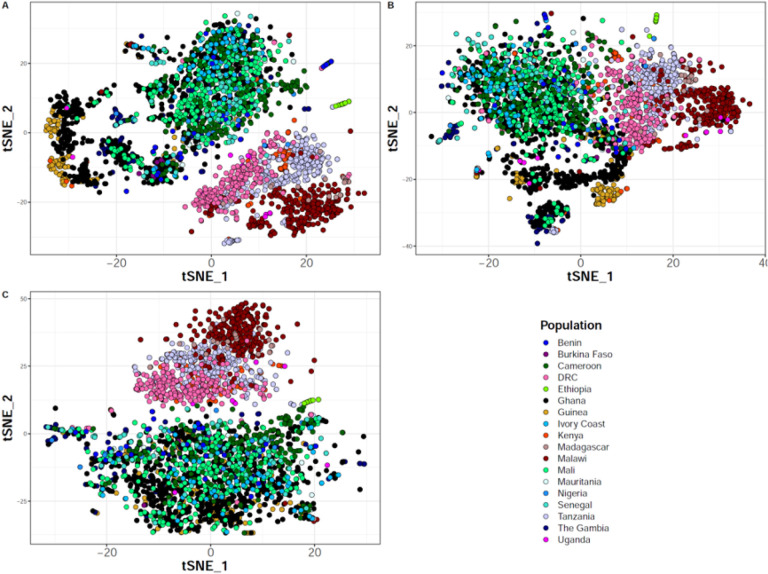
Local population structure in sub-Saharan Africa. t-distributed stochastic neighbor embedding (tSNE) was computed from the variance-standardized genetic relationship matrix generated using **A)** SNPs and indels combined, **B)** indels only and **C)** SNPs only. Variant data (from chromosome 1) were pruned for linkage disequilibrium and only samples with less than 20% missing genotypes (n = 3,008) were kept. DRC: Democratic Republic of Congo

Since the performance of indel calling was markedly improved with GATK4, we examined if the inclusion of indels improved the resolution of global and local population structure using Malariagen’s Pf6 samples. After restricting our analysis to the core genome and filtering out low quality variants; our fully optimized pipeline detected more variants in total compared to GATK3. The majority (55%) of indel positions overlapped with each other and with SNP sites and formed complex multiallelic markers (2,341,377 SNPs, 1,381,687 indels and 1,686,041 multiallelic sites in 6,626 samples for our pipeline; and 2,441,874 SNPs, 889,667 indels and 1,327,993 multiallelic sites for the GATK3 VCFs). We used high quality SNPs and indels from the core regions of chromosome 1, excluding hypervariable and subtelomeric regions, after pruning them for linkage disequilibrium and selecting high-quality samples with less than 20% missing genotypes (aiming to reduce potential false noise mostly for indels). We calculated the pairwise variance-standardized genetic relationship matrix and performed a t-distributed stochastic neighbor embedding (tSNE) analysis using SNPs and indels, individually and in combination. We observed a similar general population structure between sub-Saharan Africa, South-East Asia, South America and Papua New Guinea similarly across all three variant sets as previously reported (MalariaGEN et al., 2021; Manske et al., 2013; Miotto et al., 2015), including SNPs, indels and SNPs+Indels (Additional file 1: Figure S3). Subsequently, we selected only African samples, a more challenging population to differentiate, and performed the same relatedness analysis to look at continental population structure. SNPs showed more pronounced separation of Democratic Republic of Congo from West African samples (Fig. 3C) whereas indels provided greater differentiation of parasite populations within the same geographical regions (Fig. 3B). Specifically, with indels, the vast majority of samples from Ghana and Guinea were clearly separated from the rest of parasites from West Africa; and Tanzania, Madagascar, Kenya, Ethiopia and Malawi formed more population structure within East and South-East Africa. Interestingly, when combined both SNPs and indels led to higher resolution of population separation within and across the different African regions (Fig. 3A) compared to each of them analyzed separately.

### Increasing ploidy improves variant calling performance in unbalanced mixed infections

Given the decreased performances of both GATK4 and GATK3 in mixed infections in diploid mode in general, we tested our pipelines at ploidy 6 to improve detection of low abundance within-sample variants (due to minor low abundance parasites). Since most downstream analytic tools cannot fully process hexaploid VCFs, such as decomposing complex indels into smallest fragments prior to comparing two callsets, we limited our evaluation to SNPs. The total number of additional true positive SNPs detected by the hexaploid mode relative to the diploid one on chromosome 13 was 449, 359, 112, 102, 95 and 78 for 15%:95%, 10%:90%, 15%:85%, 20%:80%, 25%:75% and 50%:50% of IT:KH01 mixed infections, respectively (Additional file1: Table S3). For the 50%:50% mixed infection sample, the hexaploid mode did not substantially improve variant detection, confirming our hypothesis whereby the diploid mode works more accurately when there are equal proportions of individual strains in the sample. These findings suggest that the polyploid modes would be more suitable for monitoring molecular markers of malaria drug resistance or detection of low abundance strains.

To verify this, we applied our pipeline 2 at ploidy 2 and 6 on 6,626 field isolate samples from MalariaGEN’s Pf6 release that have been collected from all malaria-endemic regions worldwide (MalariaGEN et al., 2021) to analyze *k13* resistant mutations that have been reported by the World Health Organization (World Health Organization, 2020). We compared mutations found by our pipelines to those present in the public GATK3 VCF (diploid mode). As expected, the hexaploid mode of the optimized GATK4 detected 32 additional resistant mutations that were totally missed by GATK3 (Table 1). These resistant samples rescued by the hexaploid mode of our optimized pipeline were confirmed by visualizing their respective BAM files in IGV (examples are illustrated in Supplementary Figure S4 for C580Y mutation). Interestingly, one sample from Cameroon in Central West Africa where artemisinin resistance is yet to be reported, classified as wildtype by GATK3, was found with C580Y mutation (Additional file1: Figure S4).

### Our optimized pipeline with ploidy 6 enables robust estimation of complexity of infections

**Figure 4:**
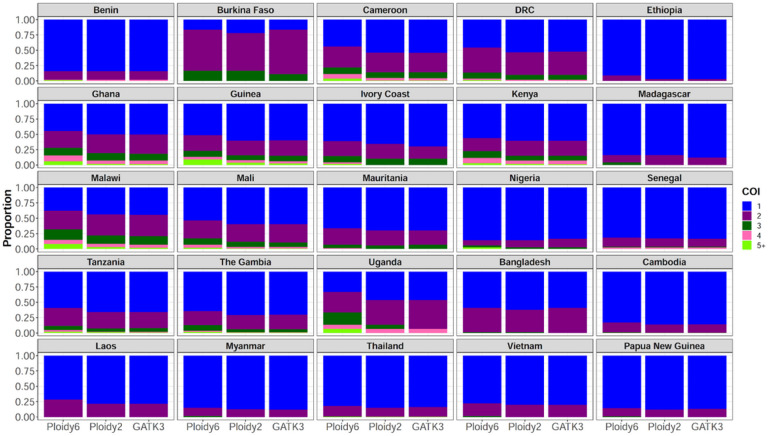
Complexity of infection analysis by site based on the optimized GATK4 pipeline with ploidy of 6 and 2 versus GATK3. This analysis included SNPs with minor allele frequencies > 1% and missingness rates < 10% on the core region of chromosome 13 from public Illumina reads of field isolate samples (n = 6,626). Ploidy 6 and ploidy 2 refer to the optimized GATK4 pipeline ran at hexaploid and diploid modes that were compared to GATK3 (publically available callset from MalariaGEN Pf6). Ploidy 6 showed significant increase in polyclonal sample detection compared to ploidy 2 and GATK3 after pairwise statistical analysis between pipelines (p < 0.05, Wilcoxon test). DRC: Democratic Republic of Congo. COI: complexity of infection.

To examine the practical benefits of improved variant calling, we assessed the overall WGS dataset complexity of infection (COI) in field isolates from sub-Saharan Africa, South-Asia Asia and South America. We computed COI based on common SNPs on chromosome 13 called using our optimized pipeline with ploidy of 6 and 2 and the GATK3 at diploid mode. Variants with minor allele frequencies > 1% and missing genotypes > 10% were selected for the COI calculation using the REAL McCOIL package (Chang et al., 2017). Overall, more mixed infections were found when the hexaploid callset was used to estimate the COI compared to the other VCFs and these results were consistent across all sampling locations although polyclonality was generally reduced outside sub-Saharan Africa (Fig. 4). We found 52.2%, 58.2% and 58.3% monoclonal infections in sub-Saharan African regions using optimized GATK4 with ploidy 6 and ploidy 2 and GATK3, respectively. Regarding biclonal infections in the same regions, we found 28.2%, 28.6% and 29.2% with our pipeline at ploidy 6 and ploidy 2 and GATK3, respectively. The prevalence of samples with COI > 2 was 19.6%, 13.2% for the hexaploid and diploid modes of the optimized GATK4, respectively and 12.5% for GATK3. Thus, increasing the ploidy up to 6 significantly increased the detection of polyclonal samples compared to ploidy 2 for GATK4 and GATK3 (p < 0.05, Wilcoxon test) in 8 sampling sites; including Cambodia, Cameroon, Democratic Republic of Congo, Ghana, Guinea, Malawi, Mali and Tanzania.

## Discussion

Our study, which aimed to adjust GATK4 settings to *P. falciparum* WGS and develop a new robust training dataset for accurate removal of low-quality variants from raw VCFs, provides an optimized variant calling pipeline that outperforms existing similar resources (MalariaGEN et al., 2021). Unlike GATK3 and default GATK4, our fully optimized pipeline is equally competent at calling SNPs and indels with high sensitivity. Thus, these results fill an important gap regarding the WGS of *P. falciparum* today and should encourage a wide use of the indels alongside the SNPs to improve the resolution of the genomic epidemiology analyses, especially within closely-related parasite populations. Importantly, our findings show that this optimized pipeline is also more robust with clinical mixed infection samples in which the number and relative frequency of each component strain is variable and undetermined. In further analysis based on field isolate samples, we demonstrate that increasing the ploidy values in HaplotypeCaller significantly improves the sensitivity of the variant calls, which might be impactful in tracking drug resistance mutations or estimating COIs, especially in hyperendemic regions in sub-Saharan Africa where artemisinin resistance is actively monitored and mixed infections are common (MalariaGEN et al., 2021; Mobegi et al., 2012).

Although GATK4 with default settings was more sensitive and precise than GATK3 (MalariaGEN et al., 2021) in general, the new training set significantly improved its performance. This achievement can be attributed not only to the sensitivity of GATK4 to keep more likely true variants from mapped reads during the initial HaplotypeCaller and GenotypeVCFs steps but also to the accuracy of the downstream soft filtering of the raw VCFs by our custom training dataset. Interestingly, modifications of parameters that control for base and mapping qualities as well as malaria-adjusted prior heterozygosities improved the sensitivity of the pipeline in longer Illumina reads from both single and mixed infection samples without significant increase in the amount of false variants and thus should work well for all current and future sequencing.

One innovation we presented is a new and simple computational method to synthesize a positive training dataset using accurate PacBio assemblies of lab strains as templates that can be reproduced in other organisms in which cross models are unavailable. This errors-depleted benchmark truth callset was able to train the Gaussian mixture model that is implemented in GATK4 to more effectively discriminate between true and false variants. Even though few assemblies were available, the size of the training dataset could be technically increased by adding mixed infection samples simulated from the unique templates with a growing number of available long-read Oxford Nanopore assemblies. Key parameters that were optimized were using a lower variant quality score log-odds (VQSLOD) threshold and increased local assembly size for haplotypecaller to better capture indels. Here we made 2kb overlapping artificial reads with 100X coverage but these key parameters can be more flexibly modified based on the goal of the variant calling project, which is hard to obtain with high precision with wet laboratory methods (Li et al., 2018; Miles et al., 2016; Zook & Salit, 2011).

Given that both SNPs and indels were equally accurate, we confidently explored the impacts of both on population genetics analyses including COI and population structure. We found that indels led to more sensitive estimation of complexity of infection, more likely due to the fact that they are more abundant than SNPs in the entire core genome of *P. falciparum* as previously demonstrated (Gardner et al., 2002; Miles et al., 2016). Similarly, SNPs and indels seem to have different impacts on population structure analysis in which the former, which is more stable, provides better separation of populations between different geographic regions but the latter, which occurs more rapidly, produces high resolution of subpopulation detection within the same areas. When combined, our study demonstrated that both variants are more beneficial in population structure analysis than using SNP only because effects seem to be additive. Indels seem to show more local inbreeding between parasites on a shorter time scale that should complement the broader geographic genetic differentiation obtained with SNPs.

As a limitation of all the GATK pipelines (mainly the non-optimized pipelines) we tested, the performance of variant calls was relatively lower in mixed than single infection samples. This general issue was partially resolved by increasing the ploidy in the HaplotypeCaller package which also allowed us to detect true artemisinin-resistant SNPs on *k13* gene that were missed by the diploid mode. Additionally, there are currently limited options for the downstream analysis of polyploid VCFs as most of the existing tools were specifically made for the diploid format.

## Conclusions

In conclusion, we provide an optimized variant calling pipeline based on GATK4 that produces high quality SNPs and indel data from monogenomic and mixed infection samples. We used the output of the pipeline in downstream population genetic analyses using publicly available WGS data to demonstrate the value of incorporating indels in such studies alongside SNPs. This pipeline should contribute to improving the quality of *P. falciparum* WGS studies and both our diploid and polyploid methods can be borrowed to analyze the genomic data of other complex microorganisms in which calling accurate variants is elusive.

## Methods

- Reference callset (“gold standard”) generation
  We aligned publicly available accurate PacBio assemblies of 10 laboratory strains 7G8, Dd2, GA01, GB4, GN01, HB3, IT, KH01, KH02 and SN01 (Otto et al., 2018; Vembar et al., 2016) in fasta format onto the P *falciparum* 3D7 reference genome (Version 3) with **minimap2** version 2.17. Mapped single chromosome-long reads were used to retain all mismatches as variants using optimized **bcftools** (version 1.13) **mpileup** and **bcftools call** commands. Variant calling was validated by visualizing VCFs and BAMs together in **IGV** (version 2.4.17) to confirm the concordance between variants and mismatches. Subtelomeric and internal hypervariable regions were removed as these regions undergo frequent exchange between chromosomes such that orthology is not maintained.
- New *in silico* training dataset generation from PacBio assemblies
  We harnessed the same 10 PacBio assemblies as templates to computationally generate a positive training dataset for GATK. **Biopython** scripts were used to make 2kb fragments of each genome after every 20 nucleotide positions and fragments were saved as fasta files. This strategy produces overlapping 2kb reads with 100X coverage from which fastq files were created by setting the quality of bases to highest quality (“~” in ASCII format) using bbmap. These reads were mapped onto the 3D7 reference genome using minimap2 (version 2.17). BAM files were sorted and duplicate reads marked using tools Picard **SortSam** and GATK **MarkDuplicatesSpark** prior to variant calling, respectively. The **HaplotypeCaller** package of **GATK** version 4.1.6.0 was used to call potential variants and create genome VCFs (gVCFs). The 10 gVCFs were combined using **GenomicsDBImport** (GATK4’s tool) before running a joint genotyping with **GenotypeGVCFs** (GATK4’s tool) within the core genome. The final VCF was compared to the reference callset for each laboratory strain using **RTG Tools** to verify the quality of the training SNPs and indels. This tool was employed for all subsequent VCF comparisons, in which our accurate reference callsets were used as baselines along with the following arguments -**decompose** and - -**squash-ploid**y that normalize complex variants into smaller constituents and ignore zygosity differences between VCFs that are compared, respectively.

### Developing an optimized variant calling pipeline

The application of an optimized pipeline to Illumina sequencing is illustrated in supplementary Figure S5. Code for the pipeline is available (https://github.com/Karaniare/Optimized_GATK4_pipeline). Illumina reads of laboratory stains and Pf6 samples were downloaded from SRA using **sratoolkit** (version 2.8.2–1). Trimmed paired reads were competitively aligned to the *P. falciparum* 3D7 and human (hg38) reference genomes with **bwa** (version 0.7.15). Reads mapping specifically onto the 3D7 genome were selected before cleaning the bam files using **CleanSam**. Clean BAM files were sorted and processed for duplicate marking using **SortSam** and **MarkDuplicatesSpark**, respectively. Mixed infection samples were simulated after combining 95%, 90%, 85%, 80%, 75% and 50% mapped reads of the KH01 strain with 5%, 10%, 15%, 20%, 25% and 50% mapped reads of IT strain to make final BAMs with 100X coverage. To optimize **HaplotypeCaller (GATK**4) for initial variant calling from *P. falciparum* WGS data with higher sensitivity, we adjusted multiple parameters as follows: -**heterozygosity** (prior SNP rate) 0.0029, -**indel-heterozygosity** (prior indel rate) 0.0017, -**min-assembly-region-size** 100, **min-base-quality-score** (minimum base quality required to consider a base for variant calling) 5 and -**base-quality-score-threshold** 12. Heterozygosity values were chosen based on SNP and indel rates calculated from the reference callsets of the 10 laboratory strains. Base quality score filtering thresholds were made less stringent to allow the algorithm to process higher amounts of reads. The mapping quality filter **(-DF** MappingQualityReadFilter) was also disabled for the same reason. Optimized HaplotypeCaller was run on each sample independently to initially detect potential variants that are stored in gVCFs. For joint genotyping option, gVCFs were first combined into a genomic database using **GenomicsDBImport**. Per-chromosome genotyping of the gVCFs was performed using the **GenotypeGVCFs** command in which -**stand-call-conf** (minimum phred-scaled confidence threshold at which variants should be called) was set to 30 to remove the majority of false positive variants that passed **HaplotypeCaller**’s filters. We enabled the -**genomicsdb-use-vcf-codec** argument in the joint genotyping script to allow for more space in info fields when annotation sizes exceed 32-bit while processing the genomic database. Since the genotyping step is computationally costly, we ran **GenotypeGVCFs** in parallel **slurm** jobs on each chromosome partitioned into 200kb segments. We used **GATK4’**s **GatherVcfs** to merge segments into single VCFs. When indels and SNPs are called at the same positions, they are recorded as multi-allelic and incorrectly treated as indels by GATK’s VCF filtering engine. To accurately filter low-quality (likely false) SNPs and indels independently, **bcftools norm** commands were used to split multi-allelic positions before applying **GATK4’**s **VariantRecalibrator** and **AppyVQSR**. The **VariantRecalibrator** analysis was performed based on **QD**, **DP**, **FS**, **SOR** and **MQ** using either the public cross data (downloaded from ftp://ngs.sanger.ac.uk/production/malaria/pf-crosses/1.0/) or our *in silico* positive training dataset made with the 10 laboratory strains. The -**lod-score-cutoff** (VQSLOD cutoff) in **AppyVQSR** was set to 0 for SNP and −2.0 for indel (due to the mapping issue of the low complexity repeat regions) to annotate low quality variants. Low quality variants (VQSLOD < 0 for SNP and VQSLOD < −2.0) were tagged with the annotation “LOW_VQSLOD” and can be used to remove them from the VCFs. We used **bcftools norm** to merge variants with the same positions into multi-allelic loci as multi-position VCFs are hard to handle in downstream analyses. The final VCFs were functionally annotated with **SnpEff** (version 5.0d). **Bcftools view** and **bcftools query** were used for all VCF subsetting and data extraction and the outputs were plotted with R Studio.

### Increased ploidy GATK4 analysis of drug resistance

We ran our optimized pipeline on Pf6 data with -**ploidy** argument of **HaplotypeCaller** set at 6 (hexaploid mode) in comparison to 2 (diploid mode). We analyzed validated *k13* mutations associated with artemisinin resistance. The annotated VCF was subset at *k13* gene locus on chromosome 13 (1,724,600 – 1,727,877) using **bcftools view** and amino acid changes and positions were extracted with **SnpEff’**s **SnpSift.jar** package. In order to identify samples that carry these mutations, we used **GATK4’**s **VariantsToTable** to extract the **GT** information along with **REF** and **POS** into a table. We applied the same approach to analyze k13 mutations in MalariaGEN’s Pf6 GATK3 VCF which was downloaded from the publicly available repository ftp://ngs.sanger.ac.uk/production/malaria/pfcommunityproject/Pf6/Pf_6_vcf/. For mutated samples that were not found in the GATK3 VCF, BAMs were visualized in **IGV** to confirm the mutations were present across multiple reads.

### Complexity of infection analysis in field isolates

We used **bcftools view** to extract SNPs with minor allele frequencies > 1% from chromosome 13 in VCFs which included 6,626 samples from West Africa, East Africa, Central Africa, Central West Africa, South-East Africa, South-East Asia West, South-East Asia East, South America, South Asia and Papua_New_Guinea. We used **bcftools +fill-tags** to add fractions of missing genotype annotations to the VCFs and **bcftools view** to select SNPs with less than 10% missingness rates. We used custom scripts based on **vcftools** to create genotype tables from diploid and hexaploid VCFs in which reference homozygous, alternate homozygous and heterozygous calls were coded as 0, 2 and 1. We finally used the genotype tables to compute discrete COIs with the **REAL McCOIL** package (Chang et al., 2017) in **jupyter notebook**.

### Population structure analysis

To analyze the population structure, we pruned variants for linkage disequilibrium and samples for missingness (> 20%). We tested SNPs and indels of chromosome 1 individually and in combination to assess the impact of both types of variants combined on the population structure. Multiallelic sites in SNP or indel-specific VCF were split and optimized **PLINK** codes were used to compute the variance-standardized genetic relationship matrix between pairs of African samples. The variance-standardized genetic relationship matrix was used to conduct tSNE analysis using the **Rtsne** package in R. We extracted the tSNE dimensions and plotted them with **ggplot2**.

## Figures and Tables

**Table 1:** Resistant k13 mutations detected by the optimized GATK4 at hexaploid and diploid modes versus GATK3. All the mutations were found in South-East Asia except for one sample carrying the C580Y allele that was collected in Cameroon.

K13 mutations	Optimized GATK4 (ploidy6)	Optimized GATK4 (ploidy2)	GATK3
**A481V**	5	5	5
**C580Y**	664	649	650
**G449A**	6	5	5
**G538V**	24	21	21
**I543T**	36	34	33
**M446I**	38	36	36
**N458Y**	17	17	17
**P441L**	28	28	28
**P553L**	24	23	23
**P574L**	30	30	30
**R539T**	65	63	62
**R561H**	24	24	24
**Y493H**	111	106	106

## Data Availability

Data and codes are available at: https://github.com/Karaniare/Optimized_GATK4_pipeline

## Abbreviations

AT: Adenine-Thymine
COI: Complexity of infection
DRC: Democratic Republic of Congo
GATK: Genomic Analysis Toolkit
Indel: Insertion-deletion
K13: Keclh 13
MalariaGEN: Malaria Genomic Epidemiology Network
POS: Position
REF: Reference
SNP: Single nucleotide polymorphism
tSNE: t-Distributed stochastic neighbor embedding
VCF: Variant call format
VQSLOD: Variant quality score log-odds
WGS: Whole genome sequencing
